# *Raphanus sativus* cv. Sango Sprout Juice Decreases Diet-Induced Obesity in Sprague Dawley Rats and Ameliorates Related Disorders

**DOI:** 10.1371/journal.pone.0150913

**Published:** 2016-03-17

**Authors:** Fabio Vivarelli, Donatella Canistro, Andrea Sapone, Gina Rosalinda De Nicola, Clara Babot Marquillas, Renato Iori, Ippazio Cosimo Antonazzo, Fabio Gentilini, Moreno Paolini

**Affiliations:** 1 Department of Pharmacy and Biotechnology, Alma Mater Studiorum-University of Bologna, Bologna, Italy; 2 Consiglio per la ricerca in agricoltura e l’analisi dell’economia agraria-Centro di ricerca per le colture industriali (CRA-CIN), Bologna, Italy; 3 Department of Veterinary Medical Sciences, Alma Mater Studiorum, University of Bologna, Ozzano dell'Emilia, Bologna, Italy; Oklahoma State University, UNITED STATES

## Abstract

**Background:**

Obesity is recognized as a leading global health problem, correlated with an increased risk for several chronic diseases. One strategy for weight control management includes the use of vegetables rich in bioactive compounds to counteract weight gain, improve the antioxidant status and stimulate lipid catabolism.

**Aim of the Study:**

The aim of this study was to investigate the role of *Raphanus sativus* Sango sprout juice (SSJ), a Brassica extraordinarily rich in anthocyanins (AC) and isothiocyanates (ITCs), in a non-genetic model of obesity (high fat diet-HFD induced).

**Methods:**

Control groups were fed with HFD or regular diet (RD). After a 10-week period, animals were assigned to experimental units and treated by gavage for 28 days as follows: HFD and RD control groups (rats fed HFD or RD and treated with vehicle only) and HFD-treated groups (rats fed HFD and treated with 15, 75 or 150 mg/kg b.w. of SSJ). Body weight and food consumption were recorded and serum lipid profile was measured (total cholesterol, triglycerides, and non-esterified fatty acids). Hepatic phase-I, phase-II as well as antioxidant enzymatic activities were assessed.

**Results:**

SSJ lowered total cholesterol level, food intake and liver weight compared with HFD rodents. SSJ at medium dose proved effective in reducing body-weight (~19 g reduction). SSJ was effective in up-regulating the antioxidant enzymes catalase, NAD(P)H:quinone reductase, oxidised glutathione reductase and superoxide dismutase, which reached or exceeded RD levels, as well as the phase II metabolic enzyme UDP-glucuronosyl transferase (up to about 43%). HFD up-regulated almost every cytochrome P450 isoform tested, and a mild down-regulation to baseline was observed after SSJ intervention.

**Conclusion:**

This work reveals, for the first time, the antioxidant, hypolipidemic and antiobesity potential of SSJ, suggesting its use as an efficient new functional food/nutraceutical product.

## Introduction

Obesity is one of the major health problems worldwide. Already in the late 90’s, the World Health Organization (WHO) announced that obesity was reaching epidemic proportions [1], becoming one of the major public health concerns [2]. The repercussions of obesity on quality of life are well documented [3]. Obesity is strongly-positively correlated with metabolic and chronic illnesses and impairment in metabolism and endocrine functions including those of sexual functioning and fertility [4–8]. Current therapeutic approaches for treating obesity are often accompanied by high rates of secondary failure and some products licensed as anti-obesity drugs have been withdrawn because of their adverse effects [9].

In the last decades, the link between obesity and oxidative stress has been extensively investigated. Increased production of oxidizing species is accepted as a critical pathophysiological mechanism in several frequent human inflammation-based diseases, many of these closely related to or concomitant with obesity [10]. It was hypothesized that systemic oxidative stress may be directly induced by obesity, probably due to excessive fat accumulation in adipose tissue, leading to adipocyte hypertrophy and elevated levels of adipocytokine [11,12]. In both cases, directly or indirectly, obesity presents a depletion of antioxidant defenses, generally showing a depressed antioxidant status [13]. A decrease in expression or activities of antioxidant enzymes such as superoxide dismutase (SOD), glutathione peroxidase (GPX) or catalase (CAT) has been reported in obese mice models [11]. Notably, the expression of enzyme GST-A4 in humans has been found to be considerably reduced in obese insulin-resistant subjects [14].

Many epidemiologic and basic studies have thus recently focused on searching for new natural lipid-lowering or antioxidant agents capable of counteracting the adverse effects induced by a high-fat diet (HFD) [15,16]. Due to their antioxidant, anti-carcinogenic, and anti-inflammatory effects, phytochemicals such as polyphenols, thiols, terpenoids and organosulfurs have been attributed several putative therapeutic roles, including those against several pathologies and chronic diseases [17]. Glucosinolates (GLs), and their relative isothiocyanates (ITCs), particularly abundant in *Brassicaceae*, have been known to act as phase II and antioxidant machinery inducers, together with the ability to scavenge reactive oxygen species as well as inhibit metabolizing phase I enzymes [18–21].

Phase I oxidation, reduction and hydrolysis, and phase II conjugation reactions, represent the multiple enzymatic pathways, mainly involved in xenobiotics metabolism and clearance [22]. Data from both animals and humans correlate obesity with an induction of some cytochrome P450 isoforms (CYPs) [21,23]. This is of particular interest if we consider that members of the CYP1, CYP2, and CYP3 families are best known for their involvement in phase-I drug metabolism and for the biotransformation of approximately 75% of all drugs in humans [24]. CYPs changes may also alter endogenous metabolism (e.g., leukotrienes, Vitamin D, arachidonic acid derivatives, nitric oxide, aldosterone, cholesterol) and some physiological functions (e.g. apoptosis, neuroendocrine functions). Finally, the existence of NADPH-dependent overproduction of reactive oxygen species (ROS) (as O_2_^-^ H_2_O_2_, and HO^.^) by animal liver microsomes has been well known for several decades [25] and has been linked to CYP induction [26]. Moreover, CYP up-regulation has been recently shown to play a key role in the advancement of steatotic liver to the non-alcoholic steatohepatitis (NASH), as well as insulin resistance development [23].

Although studies have been mainly focused on broccoli and the ITC sulforaphane, increasing evidence highlights the beneficial role of radish (e.g. Japanese radish *Daikon*), due to its higher content of ITCs and phenols [27,28]. SSJ’s chemical profile, in terms of isothiocyanate and anthocyanin content, was recently carefully characterised [29,30]. In particular, *Raphanus sativus* Sango sprouts are reported to have higher levels of ITCs than those usually found in broccoli [29]; they also contain high levels of anthocyanins (AC) [30]. ACs have been demonstrated to have beneficial effects on obesity and related metabolic complications, counteracting or delaying the occurrence of pathological states in mice fed a HFD [31–33]. Several reports also indicate that AC are able to modulate signaling pathways such as Nrf2 mediated signaling gene expression [34–36]; however, at present, no data are available on the putative health-promoting effects of Sango, and this encouraged our study.

The purpose of this study was to investigate the effects of *Raphanus sativus* cv. *Sango* freeze-dried sprout juice (SSJ) on body weight, serum lipids, xenobiotic metabolism and antioxidant enzymes in rats fed HFD.

## Materials and Methods

### Chemicals

Acetonitrile (PubChem CID:6342), aminopyrine (PubChem CID:6009), bovin serum albumin, dichlorophenolindophenol (PubChem CID:13726) (DCPIP), epinephrine (PubChem CID:5816), ethoxycoumarin (PubChem CID:35703), Folin-Ciocalteu reagent from Sigma-Aldrich, glusose 6-phosphate (PubChem CID:5958) and glucose 6-phosphate dehydrogenase from Roche Diagnostic (Indianapolis, IN, USA), L-glutathione oxidized (PubChem CID:71308714), L-glutathione reduced (PubChem CID:745), methanol (PubChem CID:5958) were HPLC grade, methoxyresorufin (PubChem CID:119220), Nicotinamide adenine dinucleotide phosphate in oxidized (PubChem CID:5886) and reduced form (PubChem CID:5886) (NADP+ and NADPH), p-nitrophenol (PubChem CID:980), pentoxyresorufin (PubChem CID:107683), phenylmethylsulfonyl fluoride, resorufin (PubChem CID:69462), sodium dithionite (PubChem CID:24489), Triton X-100, 1-chloro-2,4-dinitrobenzene (PubChem CID:6), 1-naphtol, 7-ethoxyresorufin (PubChem CID:3294) from Sigma-Aldrich.

All others chemicals were highest purity and are commercially available.

### Plant material and preparation of the freeze-dried juice

Sango seeds (Raphanus sativus (L.), cv. 0P153) were supplied by Suba Seeds Company (Longiano, FC, Italy) and stored in a dry and dark place at room temperature. Seeds were identified by a lot number and guaranteed by the producer for the quality and the homogeneity of the product. Seeds were surface-sterilised by soaking for 30 min in 1% sodium hypochlorite and rinsed with tap water. The sprouting was allowed to occur at room temperature by using an automatic sprouter VitaSeed (Suba Seeds, Longiano, FC, Italy) under an 8/16 h light/dark cycle and regular water supply, before collecting at the fourth day. Four-day old sprouts were gently washed with tap water and then squeezed using a GS1000 juice extractor (Tribest Corp., Cerritos, CA, USA) to obtain a dark violet juice, which was collected in liquid nitrogen and then lyophilised. The resulting dark violet powder was stored at -28°C prior to use.

### Animal care and treatment

All the experimental procedures were carried out in conformity with protocols endorsed by the National Academy of Science guidelines and in accordance with EU Directive 2010/63/EU for animal experiments. The protocol was approved by the Committee on the Ethics of Animal Experiments of the University of Bologna (Permit Number: 18772013). All efforts were made to minimize suffering. Thirty-five male Sprague Dawley rats (Harlan Laboratories S.r.l., Udine, Italy) aged 9 weeks and weighing 270–300 g, were housed under 12h-light/12h-dark cycle, 22°C, 60% humidity, and fed *ad libitum*. After 10-days adaptation, the rats were split randomly into five groups (7 animals per group) and fed specific diets as follows: regular diet (RD, one group, 18.7% crude protein, 5.6% crude fat, 4.5% crude fibre, by Mucedola s.r.l.); high fat diet (HFD, four groups, 23% crude protein, 34% crude fat, 5% crude fibre, by Mucedola s.r.l.). Animals maintained the dietary regimens described for 10 weeks, then each group was randomly assigned to the treatment as follows: HFD group (fed HFD and treated with vehicle only), HFD+150 group (fed HFD and treated with 150 mg/kg b.w. SSJ), HFD+75 group (fed HFD and treateted with 75 mg/kg b.w. SSJ), HFD+15 group (fed HFD and treated with 15 mg/kg b.w. SSJ), RD group (fed RD and treated with vehicle only) by gavage for twenty-eight consecutive days. The SSJ administration was always performed at the same time in the morning, far from the 12 h-dark cycle, in order to minimize the putative influence of gavage procedure on food intake. Before treatment, Sango sprout juice powder was dissolved in tap water.

### Weight gain and food intake measurement

Body weight and food consumption measurements were recorded for the duration of the experiment. Food consumption was determined for each of the five groups by weighing the total amount of food provided every day and then subtracting the amount of food still in the cages. The measurements were recorded every day and the average food consumed per rat was then obtained by dividing by the number of animals.

### Tissue collection and subcellular fraction preparation

Rats were fasted 16 h prior to sacrifice, which occurred 24 h after the last treatment. They were sacrificed by decapitation, in accordance with approved Ministerial procedures appropriate to the species. The liver was rapidly removed and processed separately. After extensive mincing with a pair of scissors, the tissue was homogenized in sucrose with an IKA Ultra-Turrax homogenizer. The S9 fraction (9,000x*g*) from the liver was then prepared [37]. The post-mitochondrial supernatant was then centrifuged for 60 minutes at 105,000x*g*, after which the cytosolic fraction (supernatant) was collected and immediately frozen in liquid nitrogen and stored at -80°C. The pellet was resuspended in 0.1 M K_2_P_2_O_7_, 1 mM EDTA buffer (pH 7.4) and centrifuged again for 60 minutes at 105,000x*g* to give the final fraction. Washed microsomes were then resuspended with a hand-driven Potter Elvehjem homogenizer in a 10 mM Tris-HCl buffer (pH 7.4) containing 1 mM EDTA and 20% (v/v) glycerol; fractions were immediately frozen in liquid nitrogen and stored at -80°C prior to use.

### Serum biochemical analyses

Blood samples were collected from each animal in both heparinized and non-heparinized tubes. Samples collected in heparinised tubes were centrifuged a tabletop centrifuge set at 2,000 rpm to obtain plasma, while samples collected in non-heparinised tubes were centrifuged at 1,500 rpm for 10 minutes after complete coagulation, to obtain serum. Biochemistry and haematology were assessed by the Department of Veterinary Medical Science, School of Agriculture and Veterinary Medicine, *Alma Mater Studiorum* University of Bologna.

### Protein concentration

The protein concentration of the microsomal and cytosolic fractions was determined according to the method described by Lowry et al. [38] as revised by Bailey [39]. Suitable concentrations and others details were previously described [40].

### Phase I enzymes

Phase I enzymes mainly represented by the superfamily of cytochrome P450, are the major enzymes involved in drug metabolism. The bioactivation of ubiquitous pre-mutagens and pre-carcinogens as well as ROS generation linked to CYPs’ activity correlate with several pathologies [41]. The enzymatic assays described below were performed on the liver microsomal fraction.

#### NADPH-(CYP)-c-reductase (CYP-red)

The analytical method is based on the determination of the reduction rate of cytochrome c at 550 nm (ε = 19.1 mM^-1^ cm^-1^), according to previously defined procedures [42].

#### Aminopyrine N-demethylase (APND)

Activity was determined by quantification of CH_2_O release, according to Mazel P [43]. The yellow colour developed by the reaction of the released of CH_2_O with the Nash reagent was read at 412 nm, and the molar absorptivity of 8,000 used for calculation [44]. The procedure was previously reported by Barillari et al. [45].

#### p-Nitrophenol hydroxylase (p-NFH)

Activity was determined according to Reinke et al. and absorbance at 546 nm was immediately recorded and 4-nitrocathecol determined (ε = 10.28 mM^-1^ cm^-1^) [46]. The method was described previously in detail [18].

#### Pentoxyresorufin O-dealkylase (PROD), ethoxyresorufin O-deethylase, (EROD) and methoxyresorufin O-demethylase (MROD)

Resorufin formation PROD was calculated by comparing the rate of increase in relative fluorescence to the fluorescence of known amounts of resorufin (excitation 563 nm, emission 586 nm) [47]. EROD and MROD activities were measured in exactly the same manner as described for the pentoxyresorufin assay, except that substrate concentration was 1.7 mM for ethoxyresorufin and 5 mM for methoxyresorufin [48]. Details of the reaction mixtures have been reported by Melega et al. [40].

#### Ethoxycoumarin O-deethylase (ECOD)

ECOD was determined by the quantification of umbelliferone formation, according to Aitio [49]. A detailed description of the assay was provided by Canistro et al. [50]

### Phase II enzymes

Phase II enzymes are mainly transferases involved in the detoxifying step of drug metabolism as well as in making drugs or toxins more water-soluble then more easily excretable forms. Phase II enzymes play a key chemopreventive role converting chemical carcinogens into inactive or less toxic compounds and preserving DNA from adduct formation [51]

#### Glutathione S-transferase (GST)

The product of the reaction of the thiol group of glutathione with the electrophilic group of CDNB was read at 340 nm (ε = 9.6 mM^-1^ cm^-1^) [52]. The assay was assessed on cytosolic fraction. The incubation mixture and the full procedure was previously described by Canistro et al. [18].

#### UDP-glucuronosyl transferase (UDP-GT)

UDP-GT was determined kinetically using the microsomal fraction and the 1-naphtol as substrate by the continuous fluorimetric monitoring of 1-naphtholglucuronide production in the presence of uridine-5’-diphosphoglucuronic acid [53]. Experiments were performed as previously described [50].

### Antioxidant enzymes

#### Catalase (CAT)

The reaction was started in a quartz cuvette, containing 50 mM-potassium phosphate buffer and cytosol sample, by adding 30 mM H_2_O_2_. The decomposition of the substrate was measured at 240 nm and catalase activity was expressed as moles of H_2_O_2_ consumed per minute per mg protein using the molar extinction coefficient of 43.6 mM^-1^ cm^-1^ [54], as previously described [40].

#### NAD(P)H:quinone reductase (NQO1)

As reported previously in detail [40], NQO1 activity was assayed spectrophotometrically at 600 nm by monitoring the reduction of the blue redox dye of 2–6 dichlorophenolindophenol (DCPIP) (ε = 9.6 mM^-1^ cm^-1^), and expressed as moles of DCPIP reduced per minute per mg protein [55].

#### Glutathione reductase (GSSG-red)

The generation of NADP^+^ from NADPH, during the reduction of GSSG, was recorded at 340 nm for 5 min at 37°C. GSSG-red activity was calculated using the extinction coefficient of 6.22 per mM x cm, and expressed as moles of NADPH consumed/min per mg protein [56]. The method was presented in extended version by Melega et al. [40].

#### Superoxide dismutase (SOD)

SOD was determined according to the Misra H.P. and Fridovich I. assay. Briefly, the activity was assayed spectrophotometrically at 320 nm by monitoring the generation of adenochrome, one of the main products of epinephrine autoxidation at pH 10.2. SOD was calculated by using the extinction coefficient of 4.02 per mM x cm, and expressed as moles of epinephrine oxidized/min per mg protein, derived by subtracting each test curves from the epinephrine autoxidation standard curve [57].

### Statistical analysis

Statistical analysis for the enzyme determination was performed using the Wilcoxon’s rank method [58]. Statistical analysis for body or liver weight, serum lipids and food intake data was performed with the two-tailed unpaired t-test. The Anderson-Darling test was performed to check the normal distribution of the data.

## Results

### Body weight-gain, food intake and liver weight

**Fig 1:**
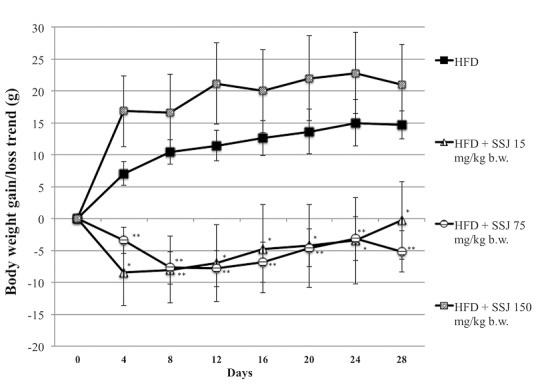
Body weight gain/loss trend of male rats with obesity induced by high fat-diet (HFD) during the Sango sprout juice (SSJ) intervention period. **Animals were treated as follows.** Rats fed HFD and treated by gavage daily for twenty-eight consecutive days with vehicle. Rats fed HFD and treated by gavage daily for twenty-eight consecutive days with 15 mg/kg b.w. SSJ. Rats fed HFD and treated by gavage daily for twenty-eight consecutive days with 75 mg/kg b.w. SSJ. Rats fed HFD and treated by gavage daily for twenty-eight consecutive days with 150 mg/kg b.w. SSJ. Values are mean ± standard error of the mean (S.E.M.) *P<0.05; **P<0.01 (two-tailed unpaired t-test).

The present study showed a strong ability of SSJ to counteract the effect of HFD diet on body weight (Fig 1). In particular, rats receiving 15 mg/kg b.w. maintained a nearly constant body weight during the SSJ intervention period, reporting a mean loss of -0.29 g against a mean of 14.67 g gained by the HFD control group (*P*<0.05). In addition, the 75 mg/kg b.w. treatment showed a significant (*P*<0.01) body weight loss (up to 5 g) compared with the HFD control group. Considering the total delta between the above-mentioned groups, the data indicated a difference greater than 19 g (*P*<0.01). The highest SSJ dose did not exert a significant change on animal body-weight, in comparison with obese rats. Daily food intake was slightly greater in the HFD control group with respect to the RD group (*P*<0.05); conversely, in the HFD + 15 mg/kg b.w. and HFD + 75 mg/kg b.w. groups, a modest but statistically significant (*P*<0.01) reduction was recorded (Table 1).

**Table 1:** Effects of *Raphanus sativus* cv. Sango sprout juice (SSJ) on bodyweight gain/loss, liver weight, and daily food intake of male rats with obesity induced by high fat-diet (HFD).

	RD		HFD		HFD + SSJ 15mg/kg b.w.		HFD + SSJ 75mg/kg b.w.		HFD + SSJ 150mg/kg b.w.	
Parameters	mean	S.E.M.	mean	S.E.M.	mean	S.E.M.	mean	S.E.M.	mean	S.E.M.
Bodyweight (g)										
Initial	429.00	10.59	516.67^††^	6.61	496.86^††^	13.56	504.14^††^	12.72	509.00^††^	18.06
Final	442.57	12.02	531.33^††^	7.41	496.57^†^	18.31	499.00^†^	14.91	530.00^††^	13.64
Bodyweight gain/loss during the intervention period (g)	13.57	2.20	14.67	2.84	-0.29**	6.07	-5.14^††^*	3.25	21.00	6.30
Food intake (g/d)	15.00	0.30	15.97^†^	0.20	14.56**	0.18	13.91**^†^	0.24	15.34	0.24

b.w. body weight; SSJ, Sango sprout juice

(Values are mean ± standard error of the mean (S.E.M.) of seven measurements performed on seven rat samples for each studied group)

Mean values were significantly different compared with the HFD group (two-tailed unpaired t-test):

*P<0.05

**P<0.01.

Mean values were significantly different compared with the RD group (two-tailed unpaired t-test):

^†^*P*<0.05

^††^*P*<0.01.

**Fig 2:**
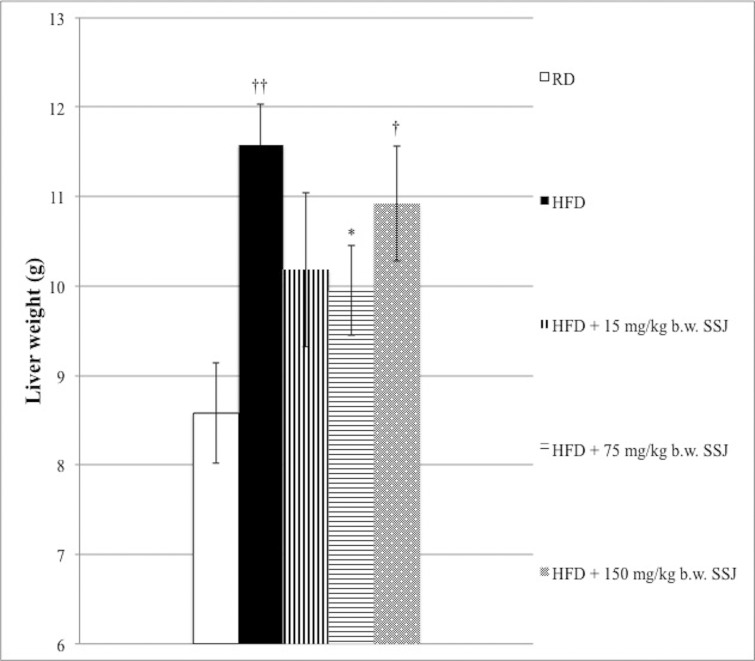
Effects of Sango sprout juice (SSJ) on liver weight of male rats with obesity induced by high fat-diet (HFD) and treated as follows. Rats fed RD and treated by gavage daily for twenty-eight consecutive days with vehicle. Rats fed HFD and treated by gavage daily for twenty-eight consecutive days with vehicle. Rats fed HFD and treated by gavage daily for twenty-eight consecutive days with 15 mg/kg b.w. SSJ. Rats fed HFD and treated by gavage daily for twenty-eight consecutive days with 75 mg/kg b.w. SSJ. Rats fed HFD and treated by gavage daily for twenty-eight consecutive days with 150 mg/kg b.w. SSJ. The bars show mean values ± standard error of the mean (S.E.M.) of seven measurements performed on seven rat samples for each studied group. Mean values were significantly different compared with the HFD group (two-tailed unpaired t-test): *P<0.05, **P<0.01. Mean values were significantly different compared with the RD group (two-tailed unpaired t-test): ^†^*P*<0.05, ^††^*P*<0.01.

**Fig 3:**
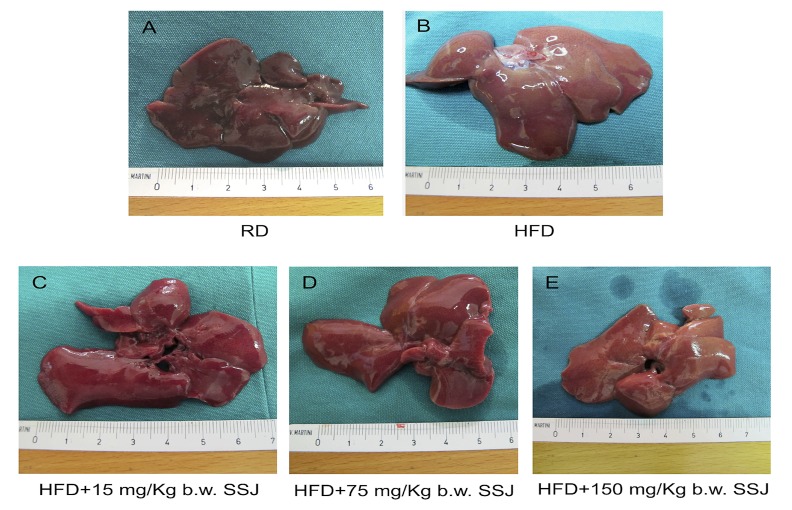
Representative macroscopic pictures of the liver from different experimental groups. **A**: Rats fed RD and treated by gavage daily for twenty-eight consecutive days with vehicle (RD). **B**: Rats fed HFD and treated by gavage daily for twenty-eight consecutive days with vehicle (HFD). **C**: Rats fed HFD and treated by gavage daily for twenty-eight consecutive days with 15 mg/kg b.w. SSJ (HFD+15 mg/kg b.w. SSJ). **D**: Rats fed HFD and treated by gavage daily for twenty-eight consecutive days with 75 mg/kg b.w. SSJ (HFD+75 mg/kg b.w. SSJ). E: Rats fed HFD and treated by gavage daily for twenty-eight consecutive days with 150 mg/kg b.w. SSJ (HFD+150 mg/kg b.w. SSJ).

As shown in Fig 2, the HFD control group showed a significant increase in liver weight (*P*<0.01) compared with RD fed rats; the build-up of fat in the liver of animals fed HFD can be observed in Fig 3. SSJ administration led to a decrease in liver weight with respect to the HFD control group, although statistical significance was reached only by the 75 mg/kg b.w. treated group (*P*<0.05).

### Serum lipid profile

**Fig 4:**
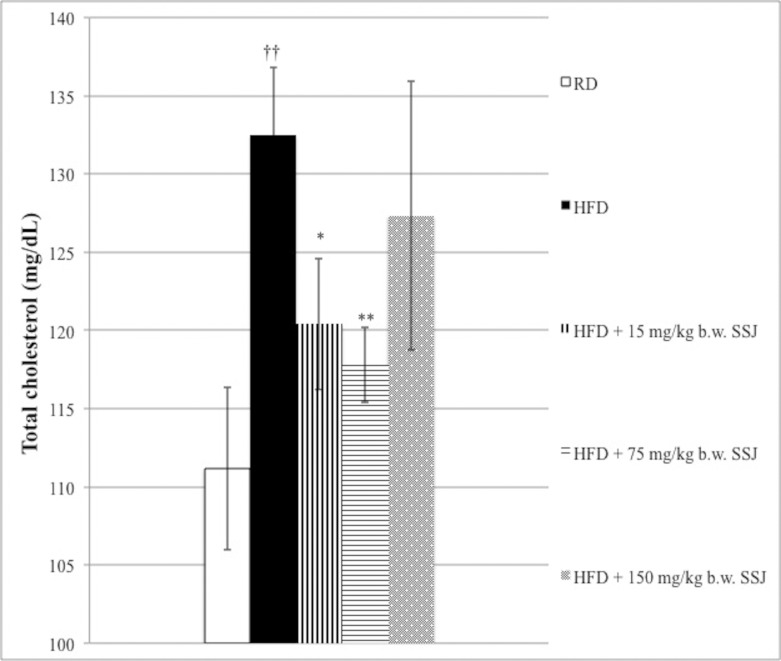
Effects of Sango sprout juice (SSJ) on cholesterol in the serum of male rats with obesity induced by high fat-diet (HFD). **Animals were treated as follows.** Rats fed RD and treated by gavage daily for twenty-eight consecutive days with vehicle. Rats fed HFD and treated by gavage daily for twenty-eight consecutive days with vehicle. Rats fed HFD and treated by gavage daily for twenty-eight consecutive days with 15 mg/kg b.w. SSJ. Rats fed HFD and treated by gavage daily for twenty-eight consecutive days with 75 mg/kg b.w. SSJ. Rats fed HFD and treated by gavage daily for twenty-eight consecutive days with 150 mg/kg b.w. SSJ. The bars show mean values ± standard error of the mean (S.E.M.) of seven measurements performed on seven rat samples for each studied group. Mean values were significantly different compared with the HFD group (two-tailed unpaired t-test): *P<0.05, **P<0.01. Mean values were significantly different compared with the RD group (two-tailed unpaired t-test): ^†^*P*<0.05, ^††^*P*<0.01.

As shown in Table 2 and Fig 4, HFD feeding significantly increased total cholesterol and triglycerides compared with the RD control group (*P*<0.05). In contrast, SSJ treatment at the lowest or medium doses produced a decrement of 12.1 and 14.7 mg/dL in total cholesterol levels (*P*<0.05; *P*<0.01, respectively). Triglycerides were not significantly affected by SSJ administration. NEFA were found lower in the HFD fed group compared with the RD control group (*P*<0.01), while a significant (*P*<0.05) increase was observed in rats that received 75 mg/kg b.w. SSJ.

**Table 2:** Effects of *Raphanus sativus* cv. Sango sprout juice (SSJ) on lipid profile in the serum of male rats with obesity induced by high fat-diet (HFD).

	RD		HFD		HFD+15mg/kg b.w. SSJ		HFD+75mg/kg b.w. SSJ		HFD+150mg/kg b.w. SSJ	
Parameters	mean	S.E.M.	mean	S.E.M.	mean	S.E.M.	mean	S.E.M.	mean	S.E.M.
TAG (mg/dL)	81.00	3.62	119.00^††^	4.92	118.40^††^	7.94	131.17^††^	9.35	138.20^††^	15.81
NEFA (mmol/L)	1.41	0.11	0.82^††^	0.05	0.87^††^	0.09	1.10**^†^	0.07	0.95^†^	0.13

b.w. body weight; TAG, Triglycerides; NEFA, non esterified fatty acids; SSJ, Sango sprout juice

(Values are mean ± standard error of the mean (S.E.M.) of seven measurements performed on seven rat samples for each studied group)

Mean values were significantly different compared with the HFD group (two-tailed unpaired t-test):

*P<0.05

**P<0.01.

Mean values were significantly different compared with the RD group (two-tailed unpaired t-test):

^†^*P*<0.05

^††^*P*<0.01.

### Hepatic phase I enzymes

Table 3 shows the activities of CYP-red and various microsomal mixed-function oxidases. It can be noted that HFD diet led to a general increase of phase I enzymes tested compared with the RD fed group. CYP2E1 linked monoxygenase was noteworthy, recording up to a 91% increase (*P*<0.01), while CYP1A2 (MROD) associated-monooxygenase showed a more modest enhancement (up to 40%, *P*<0.01); a similar increment (up to 43%, *P*<0.01) was recorded for the dealkylation of pentoxyresorufin (CYP2B1/2-linked). Again, a slight but statistically significant (*P*<0.01) enhancement was recorded for CYP3A4- and CYP1A1-supported monoxygenases (up to 11% and 10%, respectively). CYP-red was also affected by HFD, showing a 20% increase (*P*<0.01).

**Table 3:** Effects of *Raphanus sativus* cv. Sango sprout juice (SSJ) on phase I enzyme activities in the liver of male rats with obesity induced by high fat-diet (HFD).

	RD		HFD		HFD+15mg/kg b.w. SSJ		HFD+75mg/kg b.w. SSJ		HFD+150mg/kg b.w. SSJ	
Parameters	mean	S.D.	mean	S.D.	mean	S.D.	mean	S.D.	mean	S.D.
NADPH-cytochrome (P450) reductase **(CYP-red)**(nmol × mg^-1^ × min^-1^)	24.40	1.31	30.03^††^	2.29	28.76	1.35	26.24**	1.10	26.30**	1.25
Aminopyrine *N*-demethylase **APND** (nmol × mg^-1^ × min^-1^) **(CYP3A1/2)**	4.98	0.48	6.76^††^	0.44	6.20	0.20	4.32**	0.42	5.78**	0.27
p-Nitrophenol hydroxylase **pNFH** (nmol × mg^-1^ × min^-1^)**(CYP2E1)**	0.12	0.03	0.23^††^	0.02	0.25	0.05	0.24	0.03	0.13**	0.05
Ethoxycoumarin *O*-deethylase **ECOD** (nmol × mg^-1^ × min^-1^)	0.09	0.01	0.10	0.01	0.15**	0.01	0.11	0.01	0.11	0.01
Pentoxyresorufin *O*-dealkylase **PROD** (pmol × mg^-1^ × min^-1^) **(CYP2B1/2)**	6.13	0.75	10.82^††^	1.07	6.15**	0.29	4.82**	0.44	6.38**	0.55
Ethoxyresorufin *O*-deethylase **EROD** (pmol × mg^-1^ × min^-1^) **(CYP1A1)**	13.70	0.37	15.30^††^	0.67	17.90**	0.54	15.03	0.66	14.67	0.48
Methoxyresorufin *O*-demethylase **MROD** (pmol × mg^-1^ × min^-1^) **(CYP1A2)**	9.26	0.58	15.49^††^	0.55	13.42**	0.69	15.23	0.69	15.52	1.51

b.w. body weight; SSJ, Sango sprout juice

(Values are mean ± standard deviation (S.D.) of seven measurements performed on seven rat samples for each studied group)

Mean values were significantly different compared with the HFD group (Wilcoxon’s rank method):

*P<0.05

**P<0.01.

Mean values were significantly different compared with the RD group (Wilcoxon’s rank method):

^†^*P*<0.05

^††^*P*<0.01.

SSJ showed a good ability to counteract the HFD-dependent effects on the phase I enzymes described above. The most significant effect of SSJ was observed for CYP-red (up to 12% loss in 75 and 150 mg/kg b.w. groups, *P*<0.01 with respect to HFD); APND decreased with ~75 and 150 mg/kg b.w. treatments (up to 36% and 14% loss, respectively; *P*<0.01 vs HFD animals). SSJ caused a significant reduction of CYP2B1/2-suported oxidase at all doses (ranging from 41% at 150 mg/kg b.w. to 55% loss at 75 mg /kg b.w.; *P*<0.01 vs HFD group). CYP2E1 and CYP1A2 associated monooxygenase showed a decrement respectively at highest and lowest dosages (*P*<0.01). The 15 mg/kg b.w. treatment induced modest but significant (*P*<0.01) increases up to 16 and 50% for EROD and ECOD, respectively.

### Hepatic phase II enzymes

Table 4 shows the selected markers of phase II xenobiotic metabolism, GST and UDPGT. HFD diet significantly (*P*<0.01) reduced UDPGT (~30% loss) compared to RD fed rats. SSJ supplementation increased the specific activity at all doses (ranging from 43% at 75 mg/kg b.w., to 64% at 15 mg/kg b.w.) in comparison with the HFD control group (*P*<0.01). GST was not affected by the diet-induced obesity, and SSJ did not exert a significant effect with the sole exception of the lowest dosage (~19% increase; *P*<0.01).

**Table 4:** Effects of *Raphanus sativus* cv. Sango sprout juice (SSJ) on phase II enzyme activities in the liver of male rats with obesity induced by high fat-diet (HFD).

	RD		HFD		HFD+15mg/kg b.w. SSJ		HFD+75mg/kg b.w. SSJ		HFD+150mg/kg b.w. SSJ	
Parameters	mean	S.D.	mean	S.D.	mean	S.D.	mean	S.D.	mean	S.D.
Glutathione *S*-transferase (nmol × mg^-1^ × min^-1^)	10.18	0.63	10.08	0.88	12.08**	0.10	10.44	0.52	10.54	0.72
UDPglucuronosyl-transferase (nmol × mg^-1^ × min^-1^)	3.90	0.41	2.74^††^	0.29	4.50**	0.27	3.92**	0.46	4.02**	0.69

b.w., body weight; SSJ, Sango sprout juice; UDP, uridine diphosphate

(Values are mean ± standard deviation (S.D.) of seven measurements performed on seven rat samples for each studied group)

Mean values were significantly different compared with the HFD group (Wilcoxon’s rank method):

*P<0.05

**P<0.01.

Mean values were significantly different compared with the RD group (Wilcoxon’s rank method):

^†^*P*<0.05

^††^*P*<0.01.

### Hepatic antioxidant enzymes

As shown in Table 5, HFD significantly decreased (*P*<0.01) the antioxidant power compared with the RD group, recording a down-regulation of about 20% for all enzymes tested. SSJ caused a significant increment in all the treatment groups.

**Table 5:** Effects of *Raphanus sativus* cv. Sango sprout juice (SSJ) on antioxidant enzyme activities in the liver of male rats with obesity induced by high fat-diet (HFD).

	RD		HFD		HFD+15mg/kg b.w. SSJ		HFD+75mg/kg b.w. SSJ		HFD+150mg/kg b.w. SSJ	
Parameters	mean	S.D.	mean	S.D.	mean	S.D.	mean	S.D.	mean	S.D.
Catalase (μmol × mg^-1^ × min^-1^)	6.07	1.31	4.85^††^	0.30	5.80**	0.35	5.95**	0.28	5.64**	0.63
NAD(P)H:quinone reductase (nmol × mg^-1^ × min^-1^)	6.68	0.76	5.29^††^	0.58	6.65**	0.31	7.02**	0.37	5.92**	0.31
Oxidised glutathione reductase (nmol × mg^-1^ × min^-1^)	76.07	6.23	54.76^††^	6.36	76.58**	5.64	72.55**	2.91	74.61**	3.51
SOD (nmol × mg^-1^ × min^-1^)	40.02	0.30	31.87^††^	1.60	41.75**	1.18	39.60**	0.70	34.63**	1.58

b.w., body weight; SOD, superoxide dismutase; SSJ Sango sprout juice (Values are mean ± standard deviation (S.D.) of seven measurements performed on seven rat samples for each studied group)

Mean values were significantly different compared with the HFD group (Wilcoxon’s rank method):

*P<0.05

**P<0.01.

Mean values were significantly different compared with the RD group (Wilcoxon’s rank method):

^†^*P* <0.05

^††^*P* <0.01.

The complete recovery of GSSG-red and SOD at 15 mg/kg b.w. was noteworthy, exceeding the levels set at the RD regimen (*P*<0.01). The 75 and 150 mg/kg b.w. dosages also produced significant (*P*<0.01) increases of antioxidant enzymes compared with the HFD group (~33 and 36.2%, respectively for GSSG-red; ~24 and 9%, respectively for SOD). The treatment with SSJ at a dose of 75 mg/kg b.w. was able to produce a total restoration of CAT expression to the levels recorded in the RD group (*P*<0.01); the lowest and highest tested doses significantly (*P*<0.01) raised the catalytic activity if compared with the HFD group (~ 20 and 16%, respectively). An enhancement of NQO1 was observed in all intervention groups (*P*<0.01); in particular, RD levels were achieved at 15 and 75 mg/kg b.w.

## Discussion

Although several therapeutic regimens have been proposed to combat obesity and prevent the insurgence of related illnesses, at present the management of excess weight remains a highly critical issue. A large number of epidemiological studies consistently support the benefits of diets rich in fruit and vegetables for protection against various chronic diseases including obesity, dyslipidemia and related coronary heart diseases [40, 59]. Here we show a preventive role of Raphanus sativus Sango freeze-dried sprout juice, a Brassica product singly rich in AC and ITCs [29,30], against harmful outcomes induced by the obesity. To the best of our knowledge, this is the first report that shows the *in vivo* antiobesity, hypolipidemic and antioxidant effects of SSJ.

As expected, our non-genetic model showed increased BW for HFD fed rats compared with controls, that became significant from the early days of the experiment (Fig 1). After 28 days of treatment, both the low (15 mg/kg b.w.) and medium dose (75 mg/kg b.w.) of SSJ prevented BW gain but is more significant with the one dose, leading to a loss of almost 5 g in body weight (*P*<0.01) compared to an average of 14.7 g gained by the HFD group. The higher dose of SJJ did not result in weight loss or have a lipid-lowering effect; phenomena characterized by a high responses with low doses and, on the other hand, mild responses can occur with high doses, resulting in U-shaped or J-shaped curves, usually occur when plants derivatives are tested. For example, the flavonoid resveratrol displays a hormetic dose-response in a wide range of biological models [60]. We believe that the inversion trend observed in our study at the highest dose can be attributed to a similar phenomenon.

HFD induced hyperlipidemia in the animals, as confirmed by the higher serum lipid profile compared with rats fed an RD. This study revealed that SSJ supplementation was able to reduce the cholesterol which was increased by HFD, particularly at 15 and 75 mg/kg b.w dosages (Fig 4). Our results are in agreement with previous data, such as those obtained in *Moro* orange juice (rich in AC) which limited body weight gain and exerted beneficial effects on several metabolic aspects related to obesity in mice fed HFD [61]. Moreover, at the intermediate dosage, our data showed that SSJ was effective in reducing daily food intake and liver weight.

It is known that ITCs such as sulforaphane are associated with the inhibition of adipocyte differentiation by blocking clonal expansion via cell cycle arrest in 3T3-L1 preadipocytes, and stimulating lipolysis via hormone sensitive lipase activation in 3T3-L1 adipocytes [62]. Recently, it has been reported that AC treatment activates GABA_B1_R as well as decrease PKAα and p-CREB expression, suggesting the involvement of the PKA-CREB pathway in the management of obesity by anthocyanin [63]. GABA_B_ agonists, such as baclofen, showed the ability to decrease neuropeptide Y (NPY), demonstrating an anti-obesity effect as this decreases human body weight and waist circumference [64]. NPY had been shown to play a key role in appetite and food intake [65]. In addition, it is well known that the hypolipidemic properties shown by several plants are mediated by the inhibition of intestinal fat absorption, by stimulating lipid catabolism or by suppressing the fatty acid synthesis related genes (PPARγ and FAS) and inducing the expression of the β-oxidation–related gene (CPT 1) [66, 67]. Accordingly, our investigation recorded a significant NEFA increase in the 75 mg/kg b.w. SSJ supplemented group compared with the HFD animals; this trend was previously observed in rats subjected to caloric restriction [68].

In the HFD group, the hepatic antioxidant enzymes (CAT, NQO1, GSSG-red and SOD) were significantly down-regulated when compared with the control RD group. This is consistent with the literature, as inducing obesity by feeding animals with HFD led to an impairment of the antioxidant system of cells [40, 69]; similar results were also observed in human investigations [11]. SSJ administration restored hepatic enzymatic capability toward basal levels in all treated groups and even exceeded what was recorded in RD rats, where SOD and GSSG-red were more responsive (Table 3), showing an indirect antioxidant capacity, in addition to direct radical trapping properties recently described by Matera et al. [70]. These results acquired added value, if we take into account that obesity is associated with a state of chronic or low grade systemic inflammation which increases the production of obesity-related inflammatory cytokines, such as IL-1β, IL-6, TNFα, leptin and decreases anti-inflammatory cytokine levels, such as adiponectin [71, 72].

The HFD intake appears to play an important role in inducing dysregulation of drug metabolism enzymes, and thus could impair therapeutic effects or even be responsible for drug toxicity [73]. The activity of glucuronidation mediated by UDP-GT was significantly suppressed by the HFD, while SSJ was effective in restoring metabolic levels in each group. The hypothesis that some phytochemicals having a chemical structure compatible with a putative antioxidant capacity act as modulators of regulatory genes has been supported by the discovery of specific cellular pathways (Nrf2 and NF-κB regulated signalling) affected by polyphenols [74, 75]. Moreover, a recent study reported that AC were able to induce a cytoprotective effect through Nrf2-dependent gene expression [36]. We can assume that the results described here could be linked to acute activation of antioxidant and detoxifying enzymes modulated by Nrf2-ARE signalling pathway.

The significant up-regulation that occurred in almost every CYPs isoform tested in the obese rodents is noteworthy. These observations are reflected in previous studies in which it was reported that HFD could be associated with an induction of some CYPs [76]. Furthermore, obesity and chronic over-nutrition, as previously mentioned, are the major risk factors for the development of non alcoholic fatty liver disease (NAFLD) [77, 78]. The crucial role of CYP up-regulation in fat accumulation in NAFLD and in the advancement of steatotic liver to inflammatory (NASH) due to the high amount of ROS released was recently demonstrated [79]. In this frame the mild down-regulation to baseline values exerted by SSJ might be of particular interest, quite apart the crucial involvement in phase I drug metabolism in humans [24].

Taken together, these results suggest that SSJ could be a promising nutraceutical product with efficient hypolipidemic, antioxidant and antiobesity effects. Clinical investigations are obviously necessary before large-scale use of SSJ as an aid in therapeutic protocols.

## Supporting Information

S1 File ARRIVE checklist. (PDF)

## Abbreviations

AC: anthocyanins
APND: Aminopyrine N-demethylase
BW: body weight
CAT: catalase
CYP: cytochrome P-450
CYP-red: NADPH-(CYP)-c-reductase
DCPIP: dichlorophenolindophenol
EROD: ethoxyresorufin O-deethylase
ECOD: Ethoxycoumarin O-deethylase
GLs: glucosinolates
GSH: glutathione
GPX: glutathione peroxidase
GSSG: oxidised glutathione
GSSG-red: GSSG reductase
HFD: high-fat diet
ITCs: isothiocyanates
NAFLD: non-alcohol fatty liver disease
NEFA: Non Esterified Fatty Acids
NQO1: NAD(P)H:quinone reductase
p-NFH: p-Nitrophenol hydroxylase
PROD: Pentoxyresorufin O-dealkylase
RD: regular diet
ROS: reactive oxygen species
SOD: superoxide dismutase
SSJ: Sango sprout juice
TAG: triglycerides
TC: total cholesterol
